# Fine Mapping and Transcriptome Analysis Reveal Candidate Genes Associated with Hybrid Lethality in Cabbage (*Brassica Oleracea*)

**DOI:** 10.3390/genes8060147

**Published:** 2017-06-05

**Authors:** Zhiliang Xiao, Yang Hu, Xiaoli Zhang, Yuqian Xue, Zhiyuan Fang, Limei Yang, Yangyong Zhang, Yumei Liu, Zhansheng Li, Xing Liu, Zezhou Liu, Honghao Lv, Mu Zhuang

**Affiliations:** Institute of Vegetables and Flowers, Chinese Academy of Agricultural Sciences, Key Laboratory of Biology and Genetic Improvement of Horticultural Crops, Ministry of Agriculture, No. 12 Zhongguancun South Street, Beijing 100081, China; 13051379638@163.com (Z.X.); huyang@163.com (Y.H.); zxl19871009@163.com (X.Z.); xueyuqian@163.com (Y.X.); fangzhiyuan@caas.cn (Z.F.); yanglimei@caas.cn (L.Y.); zhangyangyong@caas.cn (Y.Z.); liuyumei@caas.cn (Y.L.); lizhansheng@caas.cn (Z.L.); liuxing@163.com (X.L.); liuzezhou@163.com (Z.L.)

**Keywords:** cabbage, hybrid lethality, fine mapping, transcriptome analysis

## Abstract

Hybrid lethality is a deleterious phenotype that is vital to species evolution. We previously reported hybrid lethality in cabbage (*Brassica oleracea*) and performed preliminary mapping of related genes. In the present study, the fine mapping of hybrid lethal genes revealed that *BoHL1* was located on chromosome C1 between BoHLTO124 and BoHLTO130, with an interval of 101 kb. *BoHL2* was confirmed to be between insertion-deletion (InDels) markers HL234 and HL235 on C4, with a marker interval of 70 kb. Twenty-eight and nine annotated genes were found within the two intervals of *BoHL1* and *BoHL2*, respectively. We also applied RNA-Seq to analyze hybrid lethality in cabbage. In the region of *BoHL1*, seven differentially expressed genes (DEGs) and five resistance (R)-related genes (two in common, i.e., *Bo1g153320* and *Bo1g153380*) were found, whereas in the region of *BoHL2*, two DEGs and four R-related genes (two in common, i.e., *Bo4g173780* and *Bo4g173810*) were found. Along with studies in which R genes were frequently involved in hybrid lethality in other plants, these interesting R-DEGs may be good candidates associated with hybrid lethality. We also used SNP/InDel analyses and quantitative real-time PCR to confirm the results. This work provides new insight into the mechanisms of hybrid lethality in cabbage.

## 1. Introduction

Hybrid lethality (HL), a postzygotic reproductive isolation phenomenon, plays important roles in species formation and integrity [1]. In plants, this type of genetic incompatibility occurs in the seedling or adult stage and is often associated with symptoms such as wilting, chlorosis, stunted growth, and lethality [2]. In recent years, HL cases have been reported in interspecific and intraspecific plant hybrids involving taxa such as wheat (*Triticum aestivum*), tobacco (*Nicotiana tabacum*), and cotton (*Gossypium*) [3,4,5,6]. HL not only represents a barrier to interspecific hybridization, but also remains a serious adverse event in the agricultural exploitation of wild species as a genetic resource for breeding [1,7]. Thus, studies on HL can help us better understand the evolution of species and improve crop breeding.

Several scholars have proposed hybrid lethality genetic models. These include the Dobzhansky-Muller (DM) genetic model, which is the most widely accepted model, and a dual site and unit interaction model, both of which preliminarily explain the molecular mechanism of HL [8,9]. Research on HL has been conducted in *Arabidopsis* [10,11] and lettuce [12]. This research has demonstrated that a specific allelic combination at two interacting loci is responsible for HL and that HL results from combinations of resistance (R) proteins that activate the autoimmune response, leading to lethality. These findings support the DM genetic model.

Cabbage is an important vegetable crop and is cultivated worldwide. Previously, we identified HL in cabbage, and the genetic analyses supported a DM model for the HL genes *BoHL1* and *BoHL2* [13,14]. The recent publication of the ‘02-12’ [15] and ‘TO1000’ [16] *Brassica oleracea* reference genomes has enhanced cabbage studies. Using the ‘02-12’ reference genome, Hu et al. performed genetic analysis and mapping of HL genes in cabbage and reported that *BoHL1* and *BoHL2* were on chromosome C01 of cabbage line 09-211 and chromosome C04 of cabbage line 09-222, respectively [14]. 

However, we found some problems in subsequent fine-mapping work that were likely caused by errors in the genome assembly of ‘02-12’ [17]. Thus, in the current study, we finely mapped the HL genes of cabbage based on the ‘TO1000’ reference genome using additional InDel markers and newly built segregating populations. RNA-Seq, an effective approach for detecting differentially expressed genes (DEGs) over a broad dynamic range [18], was also adopted to obtain a better understanding of the molecular basis of HL in cabbage. After combining the results of mapping and transcriptome analysis, we identified genes that are candidates for *BoHL1* and *BoHL2*, which are involved in HL in cabbage.

## 2. Materials and Methods

### 2.1. Plant Materials

Six cabbage lines were used in this study: (1) 09-211 and 09-222, carrying the HL genes *BoHL1* and *BoHL2*, respectively, were used. The hybrid plants of 09-211 and 09-222 appeared normal at the initial growth stage, but exhibited 100% seedling mortality at a later stage of growth (Figure 7A). However, the parents and F_1_ hybrids from crosses between other inbred lines (87-534, 11-196, 96-109, and 96-100) all produced normal plants. Moreover, according to the DM model, the genotypes of 09-211, 09-222, and the F_1_ hybrid (09-222 × 09-211) were ‘AAbb’, ‘aaBB’, and ‘AaBb’, respectively; (2) Four other inbred lines, 87-534, 11-196, 96-109, and 96-100, all sharing the same genotype, ‘aabb’, which do not carry any lethality genes, as confirmed by hybrid tests, were also used.

**Figure 1:**
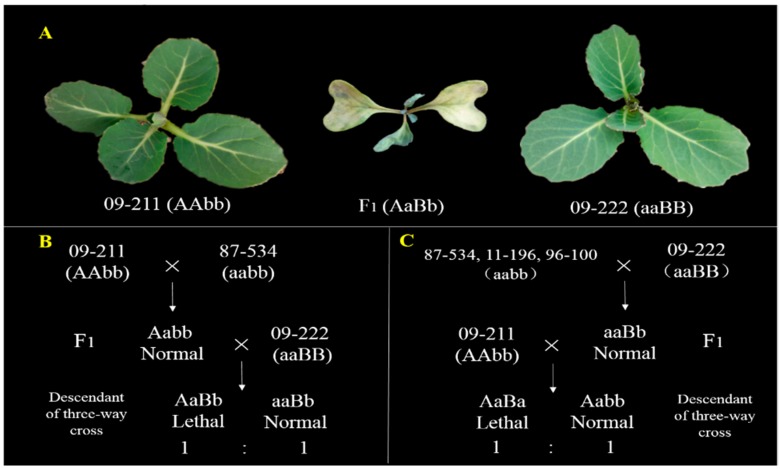
Phenotypes of the parental lines and F_1_ hybrids and the segregating population strategy. (**A**) Phenotypes of the parental lines 09-211 and 09-222 and the F_1_ hybrids; (**B**) Segregating population strategies used to map *BoHL1*; (**C**) Segregating population strategies used to map *BoHL2.*

Five descendant populations of three-way cross were employed to map the lethal genes. Two segregating populations, population A (09-211 × 87-534) × 09-222 and population B (09-222 × 87-534) × 09-211, consisting of 300 and 2855 individuals, respectively, were used to map the *BoHL1* gene. The segregating population strategies are shown in Figure 1B. The other three segregating populations, population C (09-222 × 87-534) × 09-211, population D (09-222 × 11-196) × 09-211, and population E (09-222 × 96-100) × 09-211, were generated to map the *BoHL2* gene. The segregating population strategy is shown in Figure 1C.

### 2.2. Phenotyping

The lethal symptoms in cabbage performance, e.g., retarded growth, wilting, and chlorosis, gradually appeared; most lethal individuals died at the four-leaf stage, and the remaining ones died before the rosette stage, consistent with that observed by Hu et al. [14]. According to the lethal time, we identified individuals with visually lethal symptoms at the rosette stage (approximately 30 days after germination). The segregation ratios of the descendant populations of three-way cross were analyzed with a Chi-square test using SAS software (SAS Institute, Inc., Cary, NC, USA).

### 2.3. Genotyping

#### 2.3.1. Genotyping InDel Primer Development

InDel primers were designed using re-sequencing data from 09-211 and 09-222, which were deposited into the NCBI sequence read archive (SRA) under BioSample accessions (SAMN06841129, SAMN06841130). The genomic DNA of the parent lines was subjected to whole-genome re-sequencing using the sequencing-by-synthesis method with ‘TO1000’ as the reference genome. In total, 9.3 Gb of Illumina paired-end reads were generated for both 09-211 and 09-222. Primers were designed as follows: product lengths were 150–250 bp, GC contents were 40–50%, and Tm values were 52–56 °C. Some InDel primers of previous studies [14] were also used to compare and confirm the mapping results.

#### 2.3.2. DNA Extraction

The genomic DNA of all individuals was extracted from young leaves according to the modified CTAB protocol [19]. The concentration of DNA was determined using an ND-1000 (NanoDrop Technologies, Inc., Wilmington, DE, USA) and was diluted to 40–50 ng/μL.

#### 2.3.3. Bulked Segregation Analysis (BSA)

To construct the normal and lethal pools for mapping *BoHL1*, equal amounts (50 ng/µL) of DNA from each of 12 normal and 12 lethal descendant populations of three-way cross individuals were combined into two single pooled samples for genotyping to perform the BSA [20]. The same BSA strategy was then used to map *BoHL2.* The polymorphic InDel markers between the parents were used to screen the pools, and the polymorphic markers between the pools were used to examine recombination with all of the normal individuals in the descendant populations of three-way cross.

#### 2.3.4. PCR Amplification and Electrophoresis

The 20 µL PCR mixture consisted of 4 µL of DNA template (40–50 ng/µL), 2 µL of 10 × PCR buffer, 1.6 µL of dNTP, 0.8 µL of each primer (10 µM), 0.4 µL of Taq DNA polymerase (Invitrogen, Shanghai, China) (2.5 U/µL), and 10.4 µL of ddH_2_O. The reactions were performed in a thermal cycler (Applied Biosystems Inc., Foster City, CA, USA) as follows: 94 °C for 5 min; 35 cycles of 94 °C for 30 s, 55 °C for 30 s, and 72 °C for 45 s; and a final cycle of 72 °C for 10 min. The PCR products were separated by 8% (polyacrylamide gel electrophoresis) PAGE at 160 V for 1.5 h, followed by silver staining [21].

### 2.4. Sampling, RNA Isolation and RNA-Seq

The F_1_ hybrids (09-222 × 09-211) were sown in a steady-temperature (25 °C) growth chamber (12 h light and 12 h dark photoperiod, light intensity of 65 μmol/m^2^/s^1^). Based on the lethality time, we sampled young leaves at the two-leaf stage before lethality and again when lethality had just begun to appear; the sampled leaves at these two stages were denoted ‘F1_N’ and ‘F1_W’, respectively. Three plants were randomly sampled from each treatment group, with three biological repetitions per treatment. All samples were immediately deep-frozen in liquid nitrogen and stored at −80 °C until use.

Total RNA was extracted using TRIzol reagent (Invitrogen, Carlsbad, CA, USA) according to the manufacturer’s instructions. RNA purity was assessed utilizing a NanoPhotometer spectrophotometer (IMPLEN, Westlake Village, CA, USA). RNA integrity was assessed using the RNA Nano 6000 Assay Kit for the Agilent Bioanalyzer 2100 system (Agilent Technologies, Santa Clara, CA, USA). The cDNA library preparation and sequencing were conducted by the Allwegene Technology Company in Beijing, China. All libraries were sequenced on the Illumina HiSeq 4000^TM^ platform (Illumina, Inc., San Diego, CA, USA). Clean reads were acquired after steps of raw sequence data processing. Before mapping the sequencing reads, adapter sequences were filtered from the raw reads. Low-quality reads (>50% bases with quality scores ≤5) and unknown bases (>10% N bases) were filtered from each dataset to obtain more reliable results. The Trinity program was used for the de novo transcriptome assembly of the high-quality clean reads [22].

### 2.5. RNA-Seq Data Analysis

We mapped sequencing reads to the ‘TO1000’ reference genome using TopHat [23]. To analyze and optimize the gene structure, the mapped sequencing reads were assembled and compared with known gene sequences using Cufflinks [24]. Gene expression levels were analyzed by employing the fragments per kilobase of exon model per million mapped reads (FPKM) algorithm, a commonly used method to measure the level of gene expression [25]. The DESeq package (ver. 2.1.0) [26] was employed to detect DEGs between the ‘F1_N’ and ‘F1_W’ samples. A false discovery rate (FDR)-adjusted *p*-value (*q*-value) ≤ 0.05 was used in this study as the threshold for identifying significant differences in gene expression, with biological repetitions [27]. In addition, all of the detected DEGs were compared with the non-redundant protein (NR) database [28], the cluster of orthologous groups (COG) database [29], the Kyoto encyclopedia of genes and genomes (KEGG) pathway database [30], and the Gene Ontology (GO) database [31]. Lastly, GO seq [32] and KOBAS software [33] programs were used to perform DEG functional classification and enrichment analyses to view the distribution of gene functions.

### 2.6. Fine-Mapping Results and Candidate Gene Analysis

For each marker, individuals with normal phenotypes and matching normal genotypes were recorded as ‘a’, whereas individuals with normal phenotypes but lethal genotypes were recorded as ‘h’. The Kosambi mapping function was used to calculate the genetic distances between markers [34], and the genetic map was constructed using MapDraw [35].

Since the quality of the ‘TO1000’ reference genome assembly is relatively better than that of the ‘02-12’ reference genome assembly [17], we used the former as the reference, unlike the previous mapping study by Hu et al. [14]. The genes sequence data in the mapping region were downloaded from the ‘TO1000’ reference genome, including the gene ID, gene position, and functional annotation data. These genes were also subjected to GO analysis to identify any interesting genes that might be associated with HL.

To further analyse polymorphisms of these candidate genes between 09-211 and 09-222, the Single-Nucleotide Polymorphisms (SNPs) and InDels in the coding sequence of candidate genes among 09-211, 09-222, and 96-100 were analyzed by re-sequencing data. SNPs and InDels were detected by SAMtools [36] and filtered low-quality (Phred Score < 20) reads. In the candidate genes of *BoHL1*, SNPs and InDels in the coding sequence of genes were compared between 09-211 with *BoHL1*, and 09-222 and 96-100 without *BoHL1*. Additionally, in the candidate genes of *BoHL2*, SNPs and InDels in the coding sequence of genes were compared between 09-222 with *BoHL2*, and 09-211 and 96-100 without *BoHL2.*

### 2.7. Quantitative Real-Time RT-PCR (qRT-PCR) Validation

All candidate genes were selected for validation using qRT-PCR. The gene-specific primers that were designed according to the gene sequences from the ‘TO1000’ reference genome by Primer Premier software, version 5.0 (Premier Biosoft International, Palo Alto, CA, USA) are listed in Table S1. First-strand cDNA was synthesized using the PrimeScript RT Reagent Kit (TAKARA BIO, Inc., Shiga, Japan). qRT-PCR was performed using SYBR Premix Ex Taq II (Tli RNase HPlus; TAKARA BIO, Inc., Shiga, Japan) on an ABI Prism^®^ 7900HT (Applied Biosystems, Carlsbad, CA, USA) according to the manufacturer’s instructions. The conditions for amplification were as follows: 15 min denaturation at 95 °C; 40 cycles of 95 °C for 10 s and 60 °C for 20 s; and 72 °C for 25 s. Three technical replicates were performed for each gene. The relative changes in gene expression levels were normalized to the expression of the *Medicago* actin gene (AF044573) and calculated calculated using the 2^−ΔΔCT^ method [37].

## 3. Results

### 3.1. Genetic Analysis of BoHL1 and BoHL2 with New Populations

In the autumn of 2016, the seedlings of the segregating population were cultivated in the greenhouse. F_1_ individuals of 09-211 × 87-534, 09-222 × 87-534, 09-222 × 11-196, and 09-222 × 96-100 produced normal plants, but several individuals of five descendant populations of three-way cross presented lethal symptoms (Figure 1A). In population A, 142 of 300 individuals showed lethality, and 158 showed normal growth; the lethality ratio did not differ from 1:1 (*χ*^2^_0.05_ = 0.85), as determined by the Chi-square test. Similarly, in segregating population B, 1422 of 2855 individuals showed lethality, whereas the other individuals were normal; the lethality ratio did not differ from 1:1 (*χ*^2^ = 0.04), as determined by the Chi-square test. For the other three populations segregating for *BoHL2*, i.e., populations C, D, and E, the lethality ratio was also confirmed to be 1:1, as shown in Table S2. The results confirm that the lethal phenotype in cabbage is controlled by two dominant genes: *BoHL1* from 09-211 and *BoHL2* from 09-222, in accordance with the DM model.

### 3.2. Fine Mapping of BoHL1 and BoHL2

**Figure 2:**
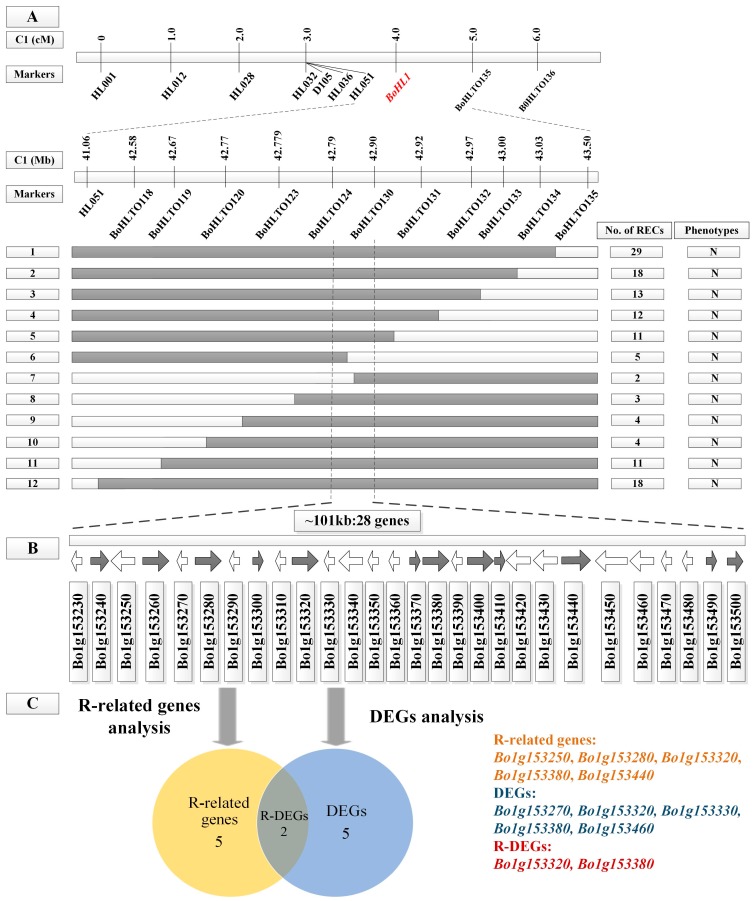
Fine mapping and analysis of *BoHL1*. (**A**) A genetic map and corresponding physical map of *BoHL1*. The *BoHL1* gene was delimited to an interval between BoHLTO124 and BoHLTO130, with an estimated length of 101 kb. N: normal phenotype; (**B**) Twenty-eight genes were annotated in the ‘TO1000’ reference genome; (**C**) R-related gene analysis and differentially expressed genes (DEG) analysis for 28 genes.

First, 50 newly designed InDel primers for chromosome C1 were used to screen the polymorphic markers linked to the *BoHL1* gene in the parents. By including some primers from Hu et al. [14], nine pairs of polymorphic InDel primers were identified in the pooled DNA samples from the normal and lethal plants. The nine primer pairs were then screened in population A. The results of screening revealed that the markers HL051 and BoHLTO135 were most closely linked to *BoHL1*, with one and one recombination events identified, respectively. Based on this mapping result, additional InDel markers were designed to identify marker loci that were closely linked to *BoHL1*. Eleven pairs of polymorphic primers were subsequently screened in population B. The InDel markers BoHLTO124 and BoHLTO130 were the markers closest to *BoHL1*, flanking the gene at genetic distances of 0.1 and 0.3 cM, respectively. A genetic map and corresponding physical map were then constructed (Figure 2A). The interval between the two markers was 101 kb (C1: 42,793,815–42,905,646 bp). This mapping result is different to that in the region of *BoHL1* reported by Hu et al. [14]; little similarity is observed between the two mapping regions. All the InDel primers used for mapping *BoHL1* are shown in Table S3A. 

**Figure 3:**
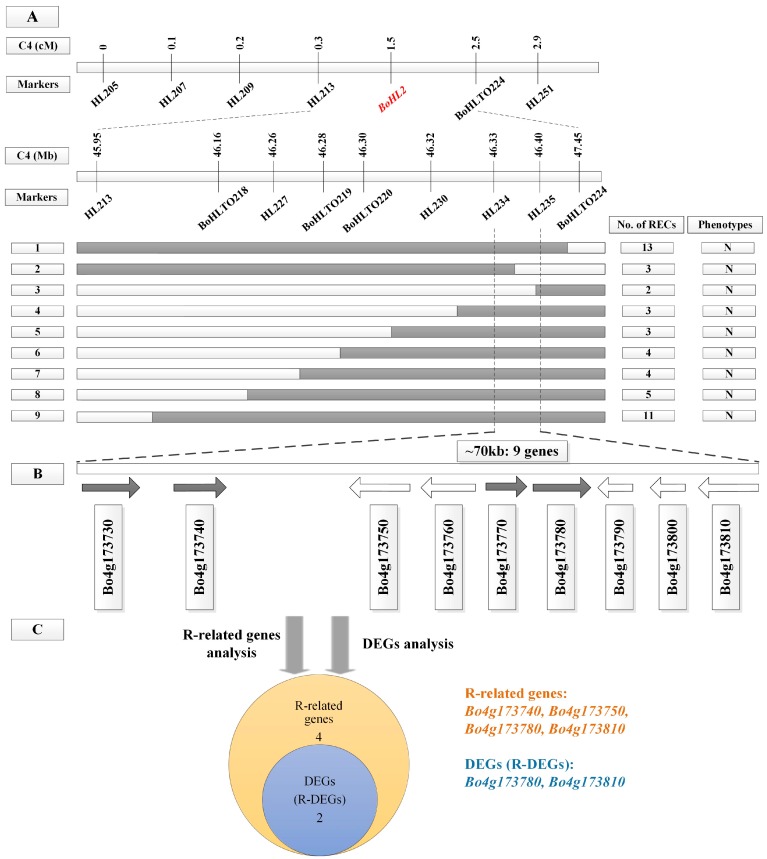
Fine mapping and analysis of *BoHL2*. (**A**) A genetic map and corresponding physical map of *BoHL2*. The *BoHL2* gene was delimited to an interval between HL234 and HL235, with an estimated length of 70 kb. N: normal phenotype; (**B**) Nine genes were annotated in the ‘TO1000’ reference genome; (**C**) R-related gene analysis and DEG analysis for nine genes.

The same strategy was used to map the *BoHL2* gene. A total of 25 pairs of InDel primers (Table S3B) on chromosome C4 showed polymorphisms, and fine mapping was performed with populations C, D, and E. The *BoHL2* gene was located between markers HL234 and HL235, which confirms the mapping position of *BoHL2* determined by Hu et al. [14]. The InDel markers HL234 and HL235 were closest to *BoHL2*, flanking the gene at genetic distances of 0.2 and 0.3 cM, respectively. A genetic map and corresponding physical map were constructed (Figure 3A). The marker interval was 70 kb (C4: 46,336,754–46,408,921).

### 3.3. RNA-Seq Data Assembly and Analysis

A total of 284,924,630 raw read pairs were generated by PE150 sequencing from six cDNA libraries (‘F1_N_1’, ‘F1_N_2’, ‘F1_N_3, F1_W_1’, ‘F1_W_2’, and ‘F1_W_3’). After quality control, 275,378,950 clean read pairs were available (Table S4). The error rates of the sequence data from the six libraries were all 1~2%. In addition, the Q20 values (i.e., reads with average quality scores >20) were all >96%, and the Q30 values (i.e., reads with average quality scores >30) were all >92%. The GC contents were all 45~47%. These results indicate that the accuracy and quality of the sequencing data were sufficient for further analysis. Approximately 70% of the clean read pairs were mapped to the ‘TO1000’ reference genome. In addition, the mapped reads were aligned to each region, including the exon, intergenic, and intron regions. The percentage of reads mapped to the exons was highest for both ‘F1_N’ (90.61%) and ‘F1_W’ (92.1%), which indicated that our reference genome was largely complete. The sequencing data has been deposited into the NCBI sequence read archive (SRA) under BioProject accession PRJNA384517 (alias: SUB2611813).

**Figure 4:**
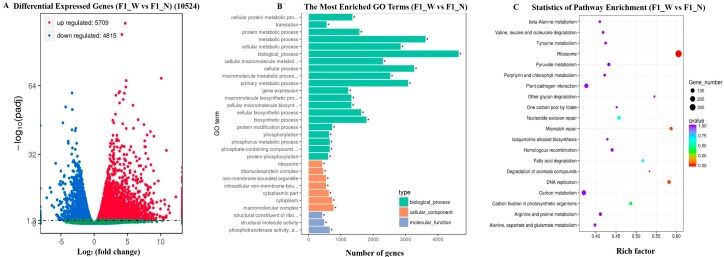
Screening of DEGs and DEG functional analysis. (**A**) Significantly up-or down-regulated genes using the *q*-value threshold of <0.05; (**B**) GO enrichment analysis of the DEGs; (**C**) The top 20 Kyoto encyclopedia of genes and genomes (KEGG) pathways with the highest representation of DEGs.

Based on the comparison of gene expression levels between the two stages, genes according to the strict ‘*q*-value < 0.05’ criterion were defined as DEGs; 10,524 (5709 up-regulated and 4815 down-regulated) DEGs of ‘F1_W vs. F1_N’ were detected (Figure 4A). The DEGs are listed in Table S5. To determine the functions of the DEGs, all 10,524 genes were subjected to GO enrichment analysis, which grouped them into three main categories (Figure 4B). In the biological process category, DEGs were highly represented by the terms ‘biological process’ (4642), ‘metabolic process’ (3620), and ‘cellular process’ (3268). In the cellular category, the most represented terms were ‘cellular component’ (2817), ‘cell’ (2696), and ‘cell part’ (2696). In addition, ‘molecular function’ (5201), ‘catalytic activity’ (2683), and ‘binding’ (2683) were among the most commonly represented molecular function terms.

All of the DEGs were analyzed using the KEGG pathway databases to identify the biological pathways. According to the richness factor value, a total of 10,524 DEGs were assigned to 122 KEGG pathways (Table S6). The pathway ‘Ribosome’ was the most common term, containing 353 DEGs, followed by ‘DNA replication’ (64), ‘Mismatch repair’ (54), ‘Carbon fixation in photosynthetic organisms’ (55), and ‘Nucleotide excision repair’. The top 20 KEGG pathways with the highest representation of DEGs are shown in Figure 4C.

**Figure 5:**
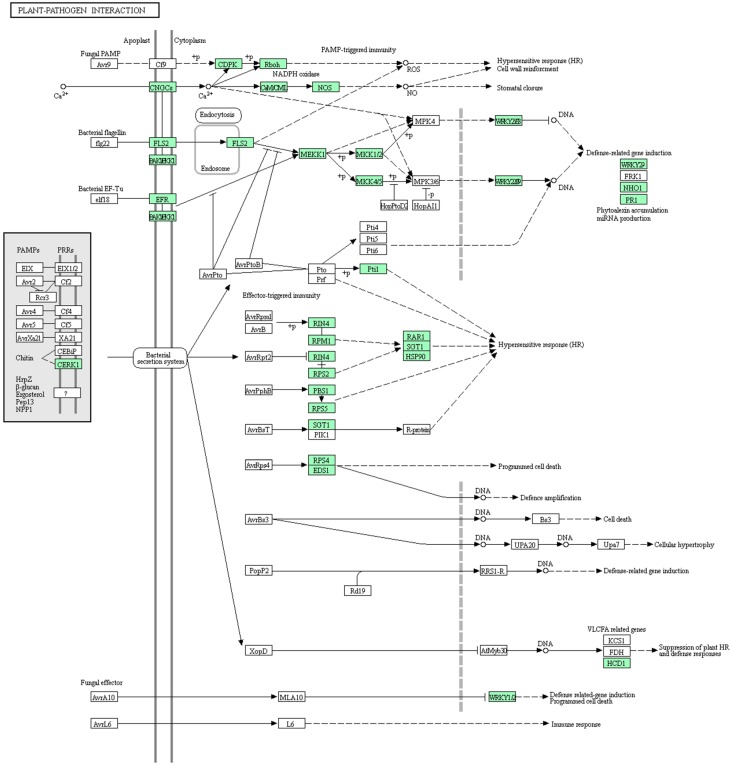
Schematic of the pathway category ‘Plant-pathogen interaction’.

Considering the opinion that HL results from combinations of resistance (R) gene products [11,12,13], we analyzed an associated term ‘Plant-pathogen interaction’ (Figure 5), which is regarded as a complex process that is controlled by the genotype and determined by the interaction of resistance genes and the corresponding virulence [38]. Moreover, it is one of the most significant in our analysis of KEGG pathway enrichment, suggesting that this pathway may play an important role in HL in cabbage. As observed in the figure, this pathway term is relevant to some complex biological processes, including the hypersensitive response (HR), programmed cell death, and immune response; some crucial genes, such as *Rin4* and *WRKY*; and R proteins. It is worth mentioning that *Rin4* is one of the two interacting genes in interspecific lettuce hybrids [12]. The *Rin4* homologous gene is *Bo3g083790*, which is an up-regulated gene, while dangerous mix 1 (*DM1*) and dangerous mix 2 (*DM2*) have a low homology in cabbage. In addition, this category supports our conclusion that HL might result from the immune response activated by combinations of R proteins. Moreover, this category may help us comprehensively analyze the close links among these processes. The DEGs in this category are shaded green in Figure 5, and a list of DEGs is provided in Table S7. Although these DEGs were not located in our mapping region, they might be downstream targets or pathways associated with the HL response.

### 3.4. Candidate Gene Analysis

#### 3.4.1. Gene Analysis for the Candidate Regions

Based on the ‘TO1000’ reference genome, 28 genes were located in the mapping region of *BoHL1*, and nine genes were located in the mapping region of *BoHL2* (Figure 2B and Figure 3B). According to ‘Gene Ontologies’ and ‘Protein Summary’ from the ‘TO1000’ reference genome, 24 of 28 genes in the mapping region of *BoHL1* and seven of nine genes in the mapping region of *BoHL2* were annotated, which are shown in Table 1 and Table 2, respectively. Considering the opinion that HL results from combinations of R gene products [11,12,13], the annotation analysis of these genes indicated that five genes in the mapping region of *BoHL1* and four genes in that of *BoHL2* are most likely related to R genes. These genes included *Bo1g153250*, *Bo1g153280*, *Bo1g153320*, and *Bo1g153380* (these genes contain the Toll/interleukin-1 receptor homology (TIR) domain and the leucine-rich repeat domain, which is the most commonly conserved domain of R proteins) [39,40]; *Bo4g173810* (the gene contains the nucleic acid-binding site (NBS), which is widely found in plant disease-resistance genes and has a high homology with genes related to apoptosis regulation) [41]; *Bo1g153440*, *Bo4g173750*, and *Bo4g173780* (these genes contain an important part of R protein, the protein kinase-like domain) [42]; and *Bo4g173740* (the gene contains the F-box domain, which is a protein-protein interaction motif) [43] (Figure 2C and Figure 3C). Thus, in total, there are nine R-related genes (five genes for *BoHL1* and four genes for *BoHL2*) that might be associated with HL in cabbage. 

#### 3.4.2. DEG Analysis for the Candidate Regions

We also analyzed the expression levels of genes in the mapping region using transcriptome data. The ‘*q*-value’ and significance of these genes in ‘F1_W vs. F1_N’ are shown in Table 1 and Table 2, respectively. Five (*Bo1g153270*, *Bo1g153320*, *Bo1g153330*, *Bo1g153380*, and *Bo1g153460*) and two (*Bo4g173780* and *Bo4g173810*) DEGs in the mapping regions of *BoHL1* and *BoHL2* were detected, respectively. Interestingly, the five DEGs of *BoHL1* were all up-regulated, and the two DEGs of *BoHL2* were all down-regulated. However, no study has shown whether the gene expression levels involved in HL are up- or down-regulated (Figure 2C and Figure 3C). Therefore, a total of seven DEGs (five genes for *BoHL1* and two genes for *BoHL2*) are interesting genes that might be involved in HL in cabbage.

As such, by combining annotation information and transcriptome analysis, in region 1, for *BoHL1*, there are seven DEGs and five R-related genes (two in common, i.e., *Bo1g153320* and *Bo1g153380*) (Figure 2C), while in region 2, for *BoHL2*, there are two DEGs and four R-related genes (two in common, i.e., *Bo4g173780* and *Bo4g173810*) (Figure 3C). Thus, we focused on all of these candidate genes (eight R genes or DEGs for *BoHL1* and four for *BoHL2*) for further analysis, especially the R-DEGs (two for *BoHL1* and two for *BoHL2*).

#### 3.4.3. Candidate Genes Analysis Using Re-Sequencing Data

In total, we identified 365 SNPs in the coding sequence of all 12 candidate genes (eight R genes or DEGs for *BoHL1* and four for *BoHL2*). Besides, no InDel was detected for them. Among the SNPs, 364 were found for the candidate genes of *BoHL1* (*Bo1g153250*, *Bo1g153270*, *Bo1g153280*, *Bo1g153320*, and *Bo1g153380*) (Table S8A); one was found for candidate genes of *BoHL2 (Bo4g173780)* (Table S8B). Moreover, 363 SNPs resulted in synonymous or non-synonymous mutations, from which we could not infer whether the gene functions were altered subsequently. However, we did find two significant SNPs in the candidate genes for *BoHL1*, including one stop-gain SNP in *Bo1g153280*: exon4: c-T1791A: p-Y597X, one stop-gain SNP in *Bo1g153380*: exon5: c-G2728T: p-E910X, which may lead to functional consequences due to protein truncation [44]. The results provide further evidence that *Bo1g153280* and *Bo1g153380* might be related to HL.

### 3.5. RNA Sequencing Validation by qRT-PCR

**Figure 6:**
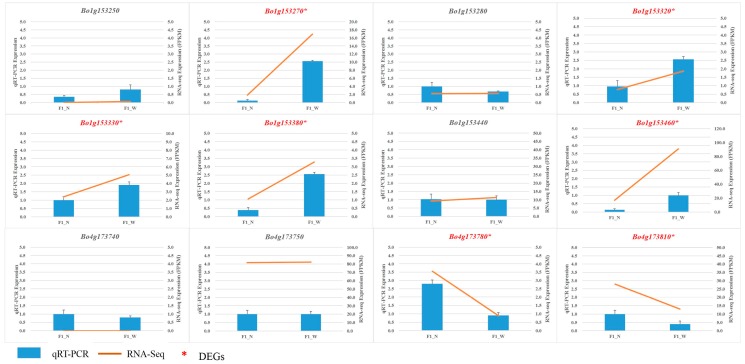
Validation of RNA-Seq data by qRT-PCR. Twelve genes were selected for validation and showed the same tendencies as revealed by the RNA-Seq data. FPKM: Fragments per kilobase of exon model per million mapped reads.

To assess the reliability of our transcriptome data, the expression fold changes of the DEGs and R-related genes (12 genes) in the mapping regions were determined using qRT-PCR and compared with those obtained using the RNA-Seq data. The FPKM values of the transcriptome data exhibited similar expression trends before and after lethal expression compared with the qRT-PCR results (Figure 6), confirming the reliability of RNA-Seq expression results. The results also confirmed that some R-DEGs might be involved in HL, such as *Bo1g153320*, *Bo1g153380*, *Bo4g173780*, and *Bo4g173810.*

## 4. Discussion

### 4.1. Omics Technologies Promote Molecular Genetics Research in B. Oleracea

The application of omics technology is becoming increasingly common and provides an opportunity to intensively study the complexity of genetic and molecular processes at the genomic, transcriptomic, proteomic, and metabolomic levels [45]. The advantages of omics technology have already been discussed elsewhere.

For example, Lv et al. reported candidate genes for cabbage *Fusarium* wilt resistance using fine mapping and RNA-Seq [46]. Kawashima et al. reported that they cloned a *Phakopsora pachyrhizi* resistance gene, *CcRpp1*, from pigeonpea (*Cajanus cajan*), and they showed that *CcRpp1* conferred full resistance to *P. pachyrhizi* in soybean by applying map-based cloning approaches and transcriptome analysis [47].

Comparative genomics analyses also allow us to identify mapping errors due to problems in genome assembly. Lee et al. demonstrated that the ‘02-12’ reference genome showed a low frequency of recombination in genetic maps and low densities of SNPs and bins on each pseudochromosome compared with the ‘TO1000’ reference genome [17]. Additionally, Liu et al. identified an incorrect insertion in the ‘02-12’ reference genome following the fine mapping and analysis of a gene conferring the glossy trait *Cgl1* in cabbage [48]. Lee et al. selected ‘TO1000’ rather than ‘02-12’ as a reference genome for their quantitative trait loci (QTL) mapping of black rot resistance because the sizes of the nine pseudochromosomes of ‘TO1000’ (446.9 Mb) are larger than those of ‘02-12’ (388.8 Mb) [49]. 

**Figure 7:**
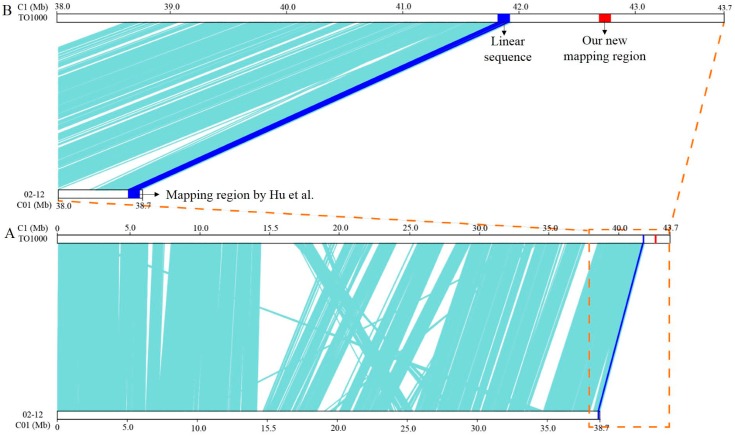
Collinearity analysis. (**A**) Collinearity analysis of the C1 chromosome in the ‘TO1000’ and ‘02-12’ reference genomes. (**B**) The physical position and collinearity analysis of the two mapping positions of *BoHL1.*

In the present study, we also encountered difficulties when fine mapping the HL genes: the markers’ positions in the linkage maps did not match their physical positions. To determine the source of the problem, we performed collinearity analysis of the C1 chromosome in the ‘TO1000’ and the ‘02-12’ reference genomes. The C1 chromosome in the ‘TO1000’ reference genome was 5 Mb larger than that in ‘02-12’, with some regions showing poor collinearity, as shown in Figure 7A. In addition, the previous mapping position of *BoHL1* was at the end of C01, based on the ‘02-12’ reference genome. Considering that the TO1000 pseudochromosomes were larger and had fewer errors, as discovered in our previous study and confirmed in the current study, we selected ‘TO1000’ for further fine-mapping study. *BoHL1* was mapped to the end of chromosome C1 (C1: 42,793,815–42,905,646 bp), which was a non-assembly chromosome part in ‘02-12’. The physical position and collinearity analysis of the two mapping positions are shown in Figure 7B. Thus, the assembly errors in the ‘02-12’ reference genome may have resulted in the incorrect mapping location of *BoHL1* by Hu et al. [14]. The ‘TO1000’ reference genome was valuable for gene mapping in the present study. Our study also represents the first application of the RNA-Seq approach to the study of HL, and we identified some DEGs related to HL. However, further work is needed to delimit the region and confirm the candidate genes by performing function analysis. 

### 4.2. Mechanisms of HL In Plants

To date, studies on HL are rare, and the molecular mechanisms remain poorly understood. Cloning of the causal genes is a crucial step to clarify these molecular mechanisms. To date, two loci causing genetic incompatibility in *Arabidopsis* have been cloned: *DM1*, a nucleotide-binding site–leucine-rich repeat (NB-LRR, NLR) gene [10], and *DM2*, a highly variable cluster of nucleotide-binding domain and leucine-rich repeat (NLR) genes [11]. Chae et al. suggested that deleterious interactions of immune receptors limit the combinations of favorable disease-resistance alleles that are accessible to plant genomes [11]. Jeuken et al. observed hybrid necrosis in interspecific lettuce hybrids and found that one of the two interacting genes was *Rin4* based on transient expression and silencing experiments [12]. This gene is one of the most important genes in the plant genome, and some responses of R genes can resist infections by pathogenic microorganisms [50]. In addition, Abramovitch et al. noted that resistance proteins that are encoded by this type of R gene can directly or indirectly induce the immune response and cause a hypersensitive response during effector-triggered-immunity (ETI) [51]. The HR, an important process in programmed cell death (PCD), plays an important role in plant disease resistance [52]. In addition, Pontier et al. argued that the HR results from the internal procedures of plant-activated genes [53]. These studies suggest that HL may be involved in the HR that is triggered by the immune response, which is tightly related to R genes.

In our current study, we further analyzed the relationship between these candidate genes and HL in cabbage. For instance, these R-DEGs (*Bo1g153320*, *Bo1g153380*) and R-related genes (*Bo1g153250*, *Bo1g153280*) encode the Leucine-rich repeat domain (LRR), and Rothberg et al. suggested that genes containing LRRs are associated with a variety of biological processes, including disease resistance, apoptosis, and the immune response in *Drosophila*, and these motifs have been implicated in protein-protein interactions as part of an extracellular domain in a variety of other proteins, which is accordant with the DM model [54]; The R-DEG *Bo1g173780* encodes the protein kinase-like domain, which is involved in a number of fundamental cellular processes such as apoptosis, proliferation, motility, and adhesion [55]. These results show that our candidate genes might been closely relevant to HL in cabbage, especially the R-DEGs. 

The present study used fine mapping and candidate gene analyses to further understand HL in plants; however, more work is needed to clarify the molecular mechanisms.

## 5. Conclusions

In conclusion, we used five descendant populations of three-way cross to finely map *BoHL1* and *BoHL2* based on the ‘TO1000’ reference genome. *BoHL1* was located on chromosome C1, as indicated by InDels BoHLTO124 and BoHLTO130, with an interval between the two markers of 101 kb. *BOHL2* was also confirmed to occur between InDel markers HL234 and HL235 on C4. We investigated the changes in transcripts involved in HL by RNA-Seq, including a gene expression level analysis and the functional enrichment of DEGs. From combining the annotation information and transcriptome analysis based on the fine-mapping results, seven DEGs and five R-related genes were found in region 1 for *BoHL1* (three in common, i.e., *Bo1g153320* and *Bo1g153380*), and two DEGs and four R-related genes were found in region 2 for *BoHL2* (also two in common, i.e., *Bo4g173780* and *Bo4g173810*). We also used SNP/InDel analyses and qRT-PCR to confirm the results. This work can inform future analyses of the functional mechanisms of HL in cabbage.

## Figures and Tables

**Table 1:** Annotations and changes in gene expression in the mapping region of *BoHL1*.

Gene ID ^a^	Gene Position on C1 ^b^	Functional Annotation ^c^	*q*-value ^d^	Significant ^e^
*Bo1g153230*	42,792,977–42,794,726	Catalytic activity	0.22844	FALSE
*Bo1g153240*	42,799,211–42,800,882	MYB domain	0.57873	FALSE
*Bo1g153250*	42,801,727–42,803,329	Toll/interleukin-1 receptor homology (TIR) domain	0.56028	FALSE
		P-loop containing nucleoside triphosphate hydrolase		
		Winged helix-turn-helix DNA-binding domain		
*Bo1g153260*	42,805,899–42,808,534	Ubiquitin-related domain	0.74850	FALSE
*Bo1g153270*	42,809,711–42,810,514	Secondary shoot formation	0.00123	TRUE
		Strigolactone biosynthetic process		
*Bo1g153280*	42,812,792–42,816,443	Toll/interleukin-1 receptor homology (TIR) domain	0.87254	FALSE
		P-loop-containing nucleoside triphosphate hydrolase		
		Leucine-rich repeat domain, L domain-like		
*Bo1g153290*	42,816,826–42,818,076	NAD(P)-binding domain	1	FALSE
		Glucose/ribitol dehydrogenase		
*Bo1g153300*	42,819,805–42,820,996	NAD(P)-binding domain	0.84117	FALSE
*Bo1g153310*	42,823,170–42,823,680	NAD(P)-binding domain	N/A	NA
*Bo1g153320*	42,828,669–42,832,299	Toll/interleukin-1 receptor homology (TIR) domain	0.00778	TRUE
		P-loop containing nucleoside triphosphate hydrolase		
		Leucine-rich repeat domain, L domain-like		
*Bo1g153330*	42,832,541–42,833,708	Oxidoreductase activity	0.00825	TRUE
		Oxidation-reduction process		
		NAD(P)-binding domain		
*Bo1g153340*	42,835,185–42,836,860	Harbinger transposase-derived protein	N/A	N/A
*Bo1g153350*	428,376,66–42,838,484	Myb-like domain	N/A	N/A
*Bo1g153360*	42,838,840–42,839,506	No comment	N/A	N/A
*Bo1g153370*	42,843,786–42,845,295	Agenet domain, plant type	N/A	N/A
*Bo1g153380*	42,846,297–42,849,389	Toll/interleukin-1 receptor homology (TIR) domain	1.43E–07	TRUE
		Ploop-containing nucleoside triphosphate hydrolase		
		Winged helix-turn-helix DNA-binding domain		
		Leucine-rich repeat domain, L domain-like		
*Bo1g153390*	42,849,475–42,850,408	NAD(P)-binding domain	0.04255	FALSE
		Glucose/ribitol dehydrogenase		
*Bo1g153400*	42,852,450–428,54,117	Armadillo-type fold	N/A	N/A
*Bo1g153410*	42,854,711–42,855,688	No comment	0.96769	FALSE
*Bo1g153420*	42,858,540–42,861,395	T-complex protein 1, theta subunit	0.40463	FALSE
*Bo1g153430*	42,862,213–42,864,216	YTH domain	0.93226	FALSE
*Bo1g153440*	42,869,749–42,873,374	Protein kinase-like domain	0.09861	FALSE
*Bo1g153450*	42,881,692–42,883,434	NAD(P)-binding domain	0.74443	FALSE
*Bo1g153460*	42,891,683–42,892,718	No comment	2.55E–32	TRUE
*Bo1g153470*	42,894,992–42,896,857	Thioredoxin-like fold	0.53912	FALSE
*Bo1g153480*	42,898,263–42,899,388	No comment	0.87865	FALSE
*Bo1g153490*	42900,367–42,900,597	Response to auxin	0.92167	FALSE
*Bo1g153500*	42,901,824–42,905,154	GDP-fucose protein O-fucosyltransferase	0.25833	FALSE

^a^ Genes ID (in ‘TO1000’) in the candidate region; ^b^ The physical positions of 28 genes on chromosome C1 (in ‘TO1000’); ^c^ Functional annotation of 28 genes; ^d^ *q*-value of 28 genes. N/A: no expression of the gene was detectable; ^e^ TRUE: significantly different; FALSE: no significant difference.

**Table 2:** Annotations and changes in the expression of genes in the mapping region of *BoHL2*.

Gene ID ^a^	Gene Position on C4 ^b^	Functional Annotation ^c^	*q*-value ^d^	Significant ^e^
*Bo4g173730*	46,336,886–46,339,234	Biosynthetic process	*0.44732*	*FALSE*
		Catalytic activity		
*Bo4g173740*	46,341,684–46,342,808	F-box domain	N/A	N/A
*Bo4g173750*	46,364,288–46,367,249	Protein kinase-like domain	0.69186	FALSE
*Bo4g173760*	46,369,761–46,371,466	Protein of unknown function DUF688	0.37224	FALSE
*Bo4g173770*	46,376,730–46,377,865	Domain of unknown function DUF1985	N/A	N/A
*Bo4g173780*	46,383,190–46,387,379	Protein kinase-like domain	3.13E–08	TRUE
		Serine-threonine/tyrosine-protein		
		Kinase catalytic domain		
*Bo4g173790*	46,388,006–46,389,868	No comment	0.13635	FALSE
*Bo4g173800*	46,395,063–46,395,976	No comment	0.07896	FALSE
*Bo4g173810*	46,405,666–46,407,281	Nucleic acid-binding, OB-fold	0.00004	TRUE

^a^ Genes ID (in ‘TO1000’) in the candidate region; ^b^ The physical positions of nine genes on chromosome C4 (in ‘TO1000’); ^c^ Functional annotation of nine genes; ^d^ *q*-value of nine genes. N/A: no expression of the gene was detectable; ^e^ TRUE: significantly different; FALSE: no significant difference.

## Supplementary Materials

The following are available online at www.mdpi.com/2073-4425/8/6/147/s1. Table S1 Information on the primers used for qRT-PCR; Table S2 Chi-square goodness-of-fit test ratios of segregating populations; Table S3 Polymorphic primers used for fine map. (A) Polymorphic primers used for *BoHL1* fine mapping. (B) Polymorphic primers used for *BoHL2* fine mapping; Table S4 Sequencing and assembly statistics for the six transcriptome datasets in ‘F1_N’ and ‘F1_W’; Table S5 List of DEGs from ‘F1_W vs. F1_N’. Listed are 10,524 (5709 up-regulated and 4815 down-regulated) DEGs of ‘F1_W vs. F1_N’; Table S6 122 List of the KEGG pathways of DEGs; Table S7 DEGs in the pathway category ‘Plant-pathogen interaction’; Table S8A the SNPs in the coding sequence of candidate genes for *BoHL1* among 09-211, 09-222 and 96-100; Table S8B the SNPs in the coding sequence of candidate genes for *BoHL2* among 09-211, 09-222 and 96-100.

## Abbreviations

HL	Hybrid lethality
DM	Dobzhansky-Muller
InDel	Insertion-deletion
SNP	Single nucleotide polymorphisms
COG	Cluster of orthologous groups
KEGG	Kyoto encyclopedia of genes and genomes
qRT-PCR	Quantitative real-time RT-PCR
DEGs	Differentially expressed genes
BSA	Bulked segregant analysis
TIR	Toll/interleukin-1 receptor homology
QTL	Quantitative trait loci
PAGE	Polyacrylamide gel electrophoresis
NBS-LRR	Nucleotide binding site-leucine rich repeat
ETI	Effector-triggered-immunity
HR	Hypersensitive response
PCD	Programmed cell death
SNP	Single nucleotide polymorphism
FPKM	Fragments per kilobase of exon model per million mapped reads
*DM1*	Dangerous mix 1
*DM2*	Dangerous mix 2
